# Supplementary figures and images for: Transcriptomic and Phenomic Investigations Reveal Elements in Biofilm Repression and Formation in the Cyanobacterium Synechococcus elongatus PCC 7942
Source: Front Microbiol. 2022 Jun 23;13:899150. doi: 10.3389/fmicb.2022.899150 (PMC9260433; doi:10.3389/fmicb.2022.899150)

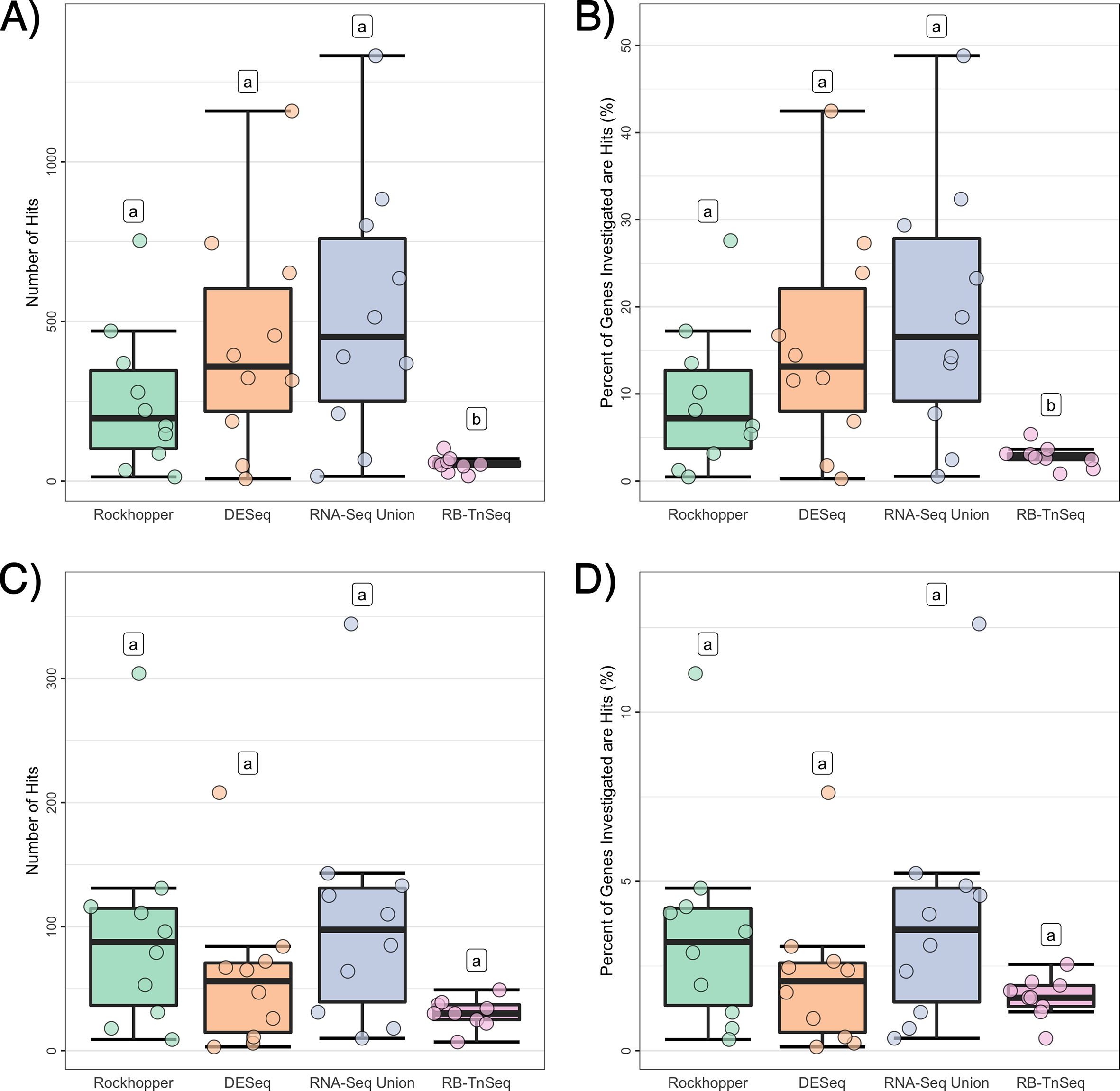

Supplement: Supplementary Figure S1 — Comparing the results of RNA-Seq and RB-TnSeq analyses. Combined scatter and box plots of (A) the number of DEGs for the pairwise comparisons presented in figure or the number of hits from all RB-TnSeq fractions, (B) the percent of genes investigated in each experiment that are DEGs or RB-TnSeq hits, (C) the number of sDEGs or RB-TnSeq hits, and (D) the percent of genes investigated in each experiment that are sDEGs or RB-TnSeq hits. Boxed letters indicate groups that are not significantly different (p > 0.05) based on pairwise two-tailed t-tests assuming unequal variances; RB-TnSeq results are significantly different from those of RNA-Seq when considering all DEGs, but not when limiting the analysis to sDEGs. [file Image_1.TIF]

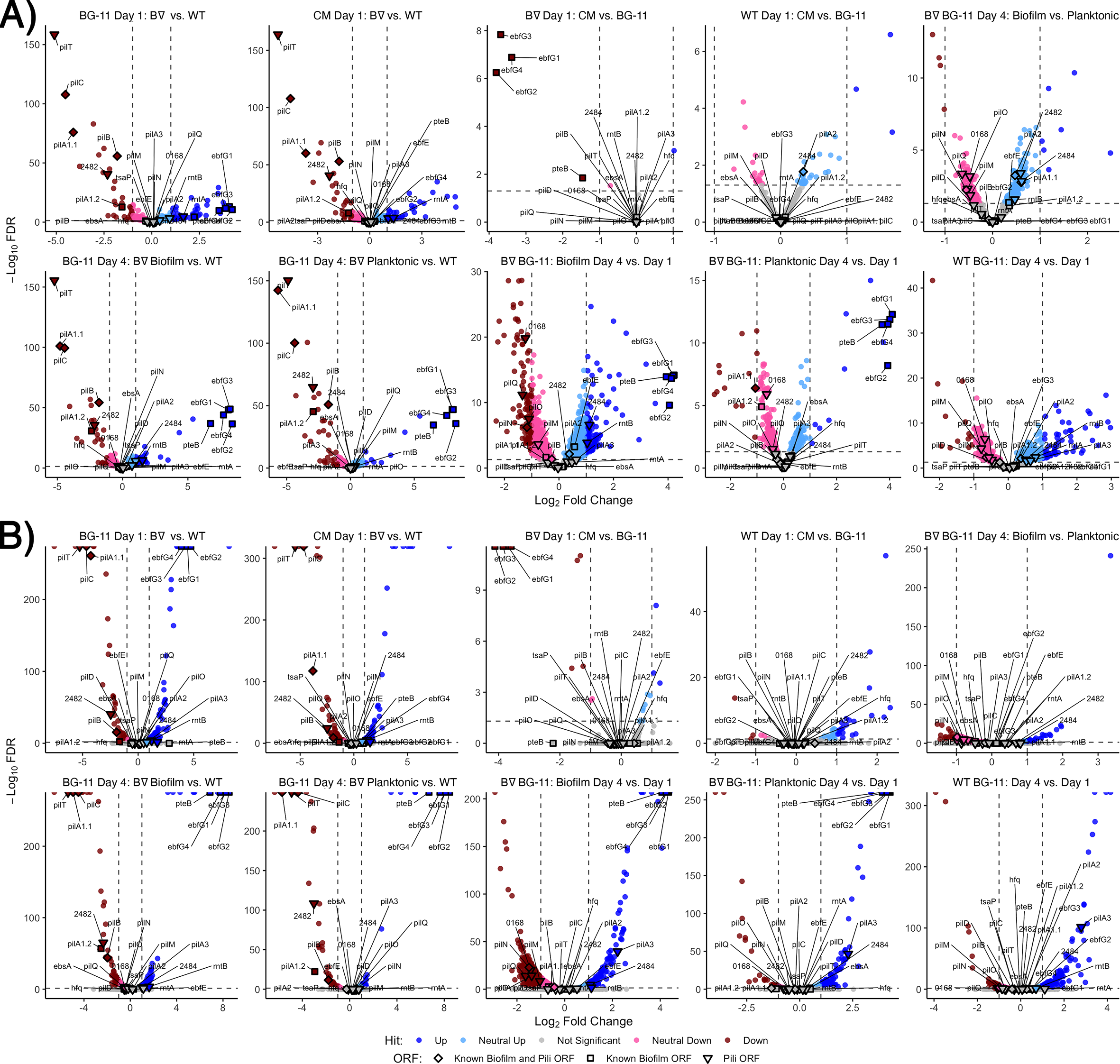

Supplement: Supplementary Figure S2 — Volcano plots for all 10 pairwise comparisons relevant to the tested RNA-Seq experimental variables. Plots are presented as described in Figures 1B,C and are derived from either the (A) DESeq analysis or (B) Rockhopper analysis of RNA-Seq data. For the Rockhopper analysis, the software returns FDR values of 0.0 for a number of ORFs, which become infinite values on the y-axis’ log-scale. These infinite data points are accumulated at the top of the graph. [file Image_2.TIF]

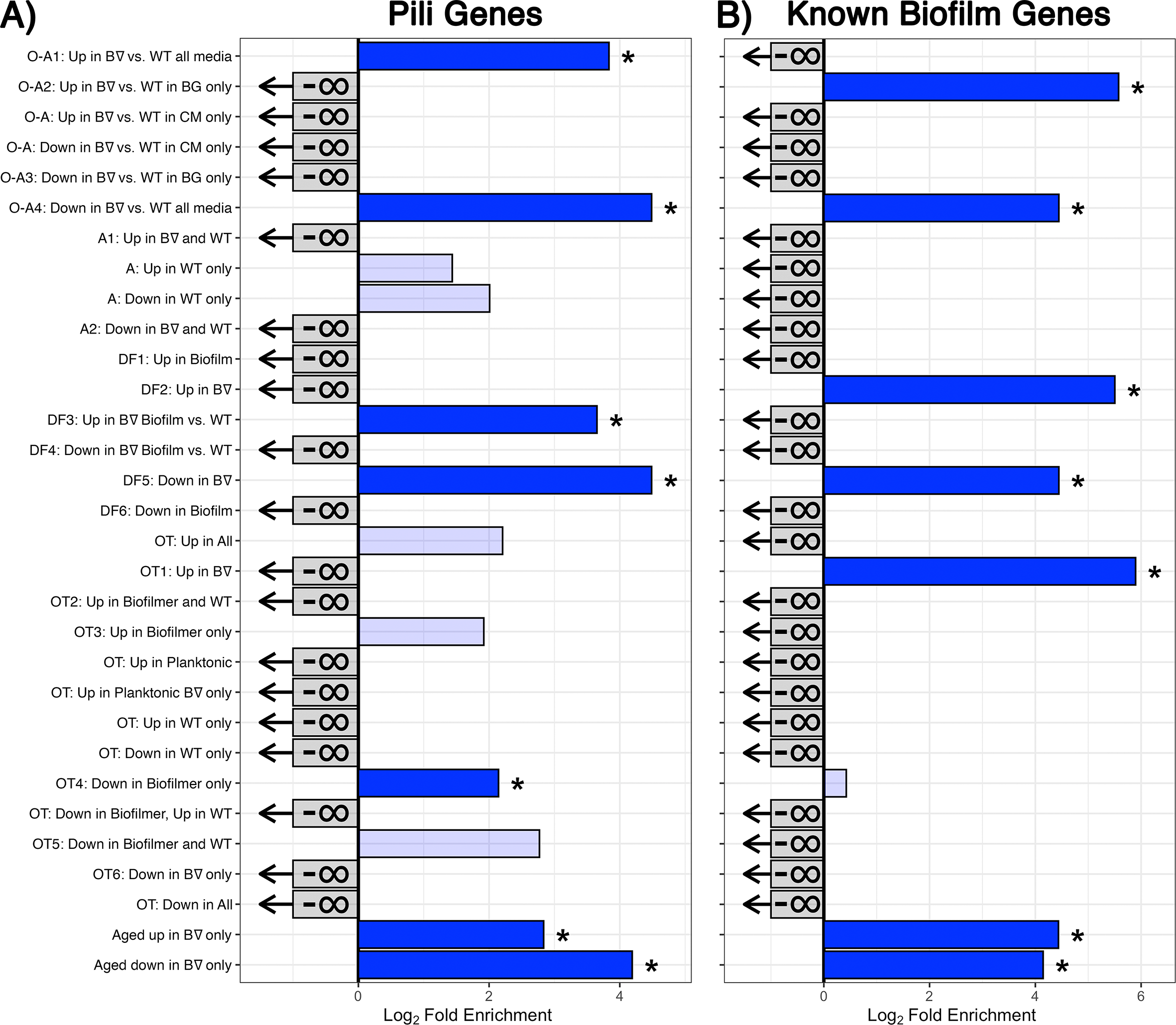

Supplement: Supplementary Figure S3 — Enrichment analyses of (A) pili genes and (B) known biofilm genes in all named RNA-seq clusters of interest, as identified in Figures 1, 2 and Supplementary File S3. The graphs are presented as described for Figure 1E. [file Image_3.TIF]

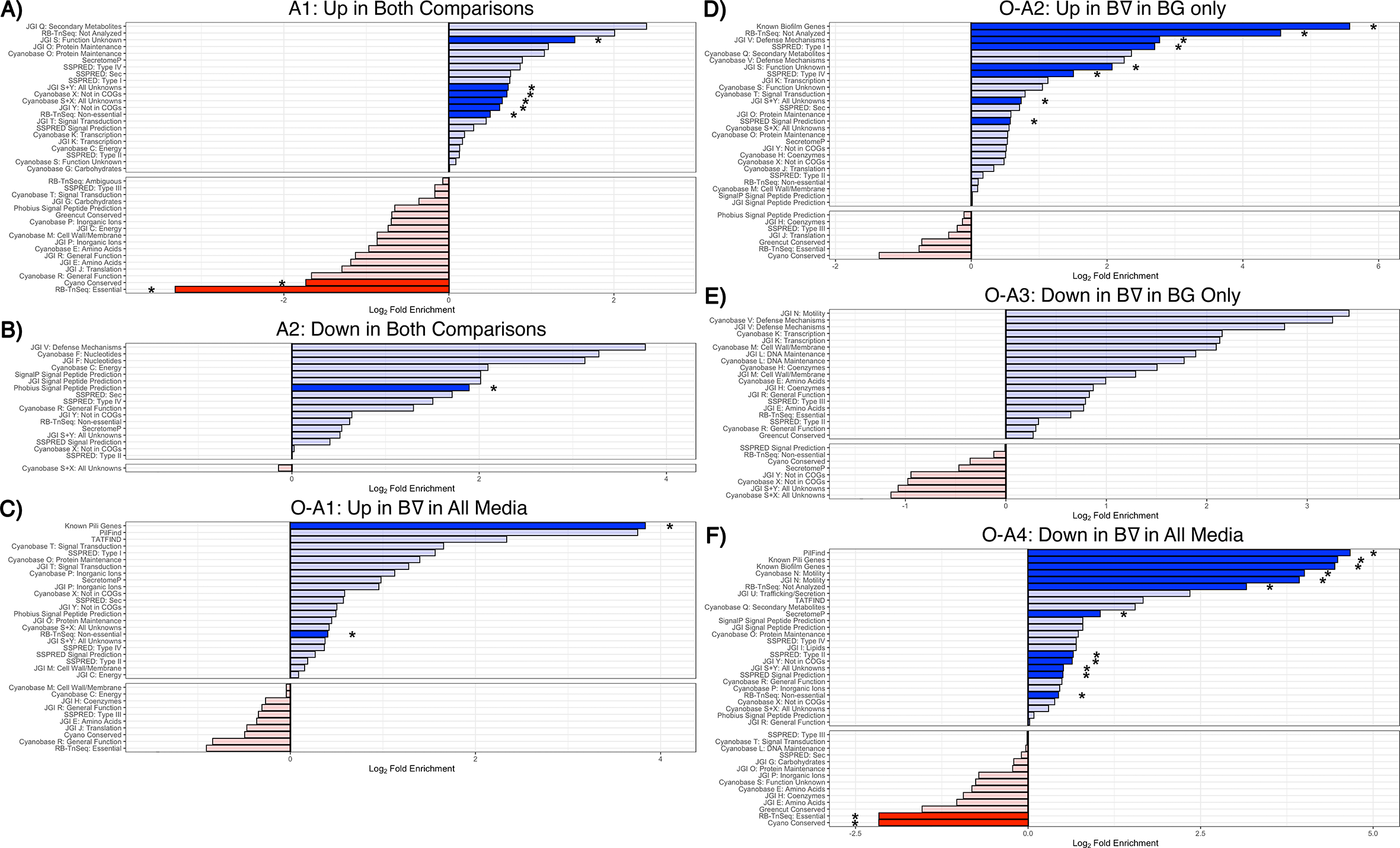

Supplement: Supplementary Figure S4 — Enrichment analyses of sDEGs groups, as identified in Figure 1D. The graphs are presented as described for Figure 1E, except only categories of information accumulated in Supplementary Table S3 with at least one gene present in the interest group are shown. [file Image_4.TIF]

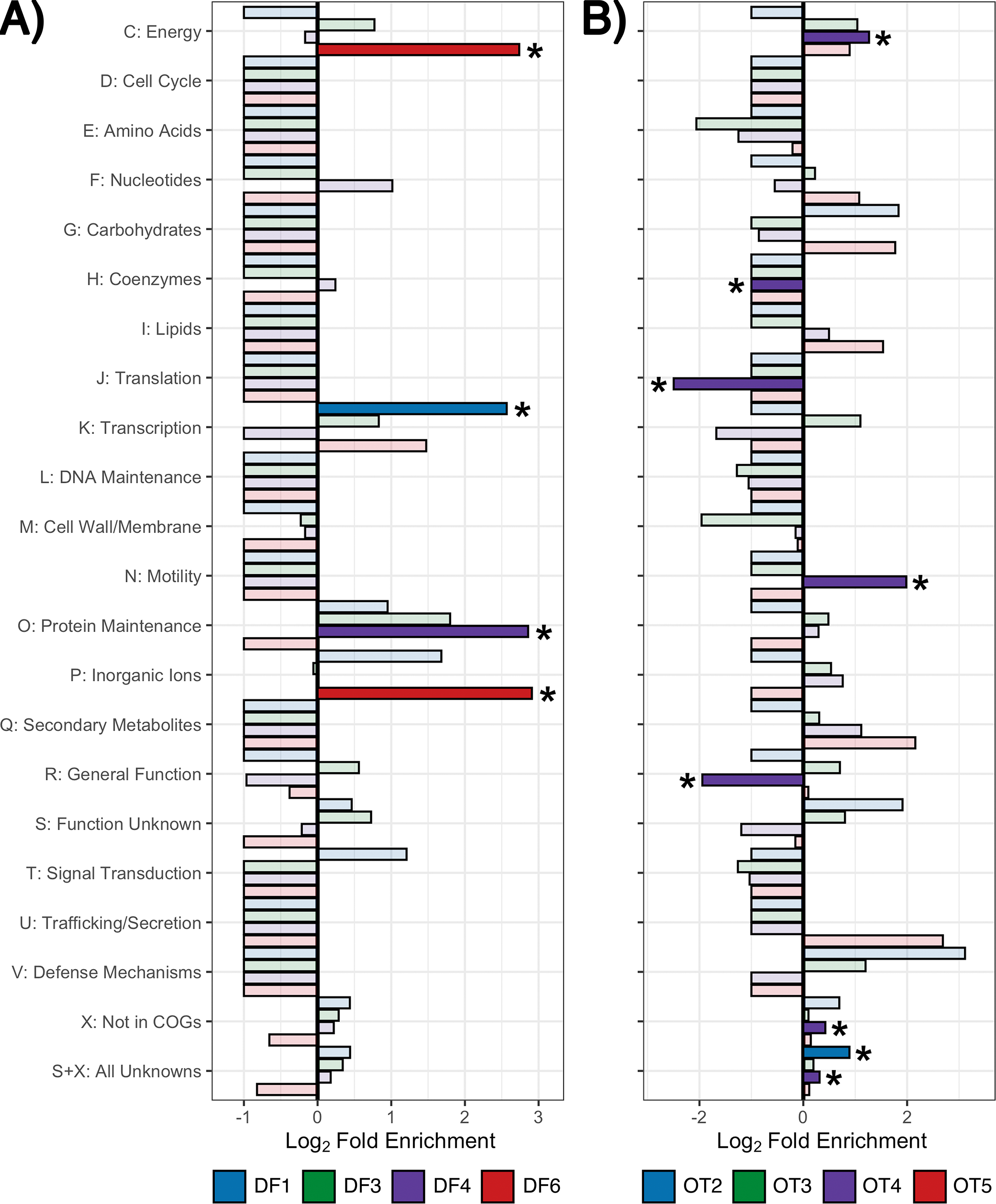

Supplement: Supplementary Figure S5 — Day 4 and time course RNA-Seq enrichment analyses. Functional enrichment analysis of Cyanobase COGs for (A) Day 4 and (B) over time groups of interest as identified in Figures 2D,E. Colors identify the data for the appropriate group of interest, as provided in the graph legend. [file Image_5.TIF]

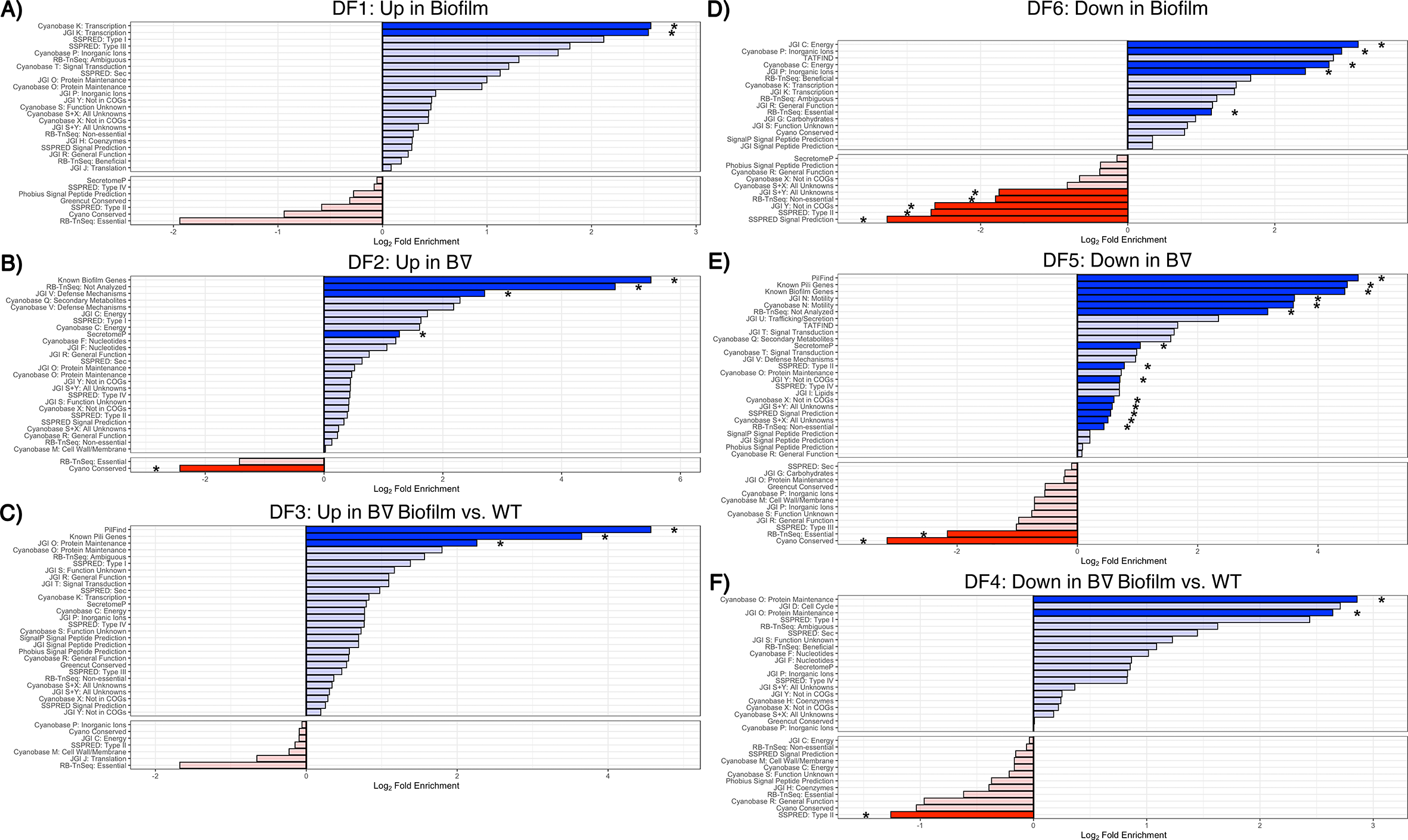

Supplement: Supplementary Figure S6 — Enrichment analyses of all Day 4 sDEGs groups, as identified in Figure 2D. The graphs are presented as described for Figure 1E, except only categories of information accumulated in Supplementary Table S3 with at least one gene present in the interest group are shown. [file Image_6.TIF]

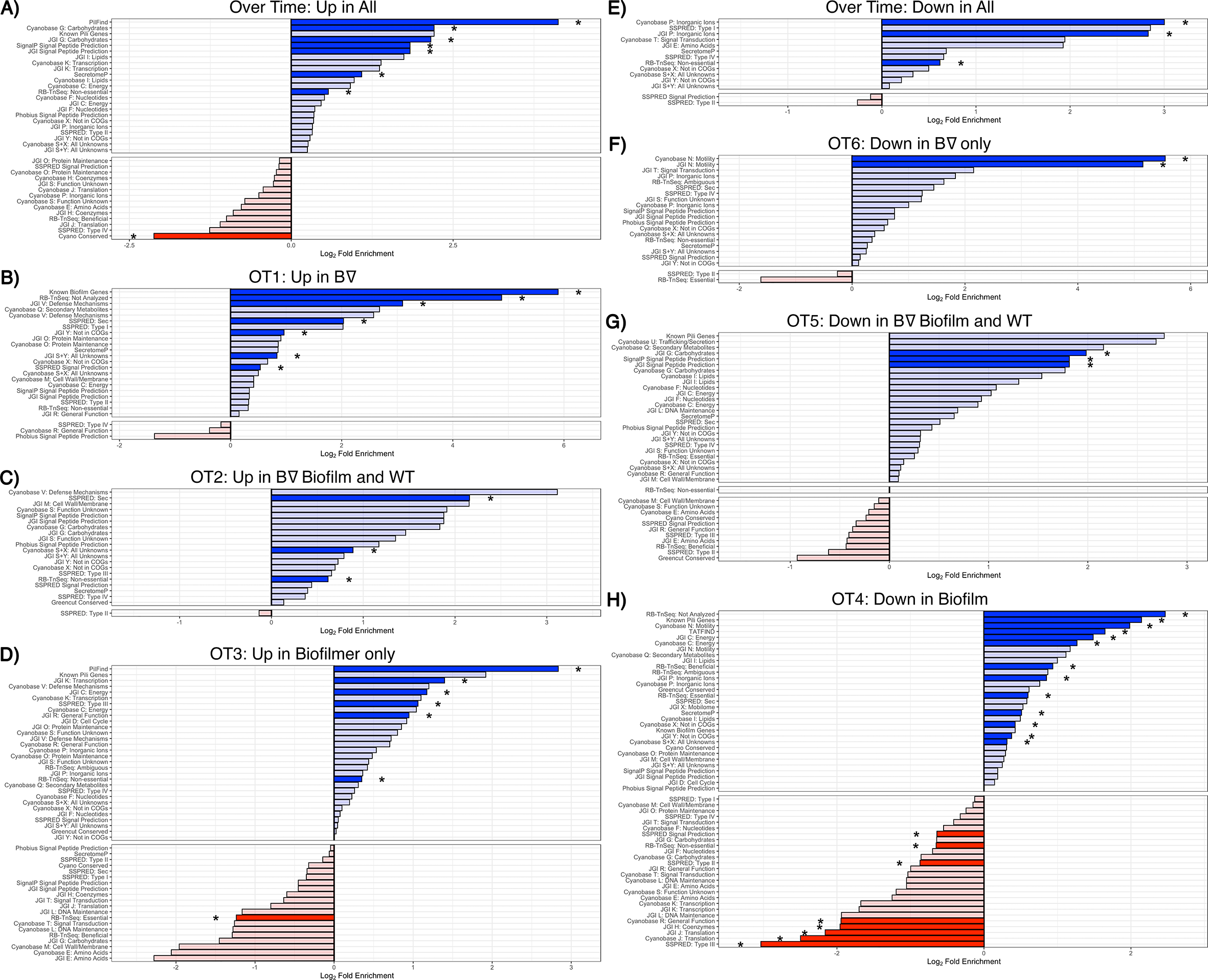

Supplement: Supplementary Figure S7 — Enrichment analyses of all over time sDEGs groups, as identified in Figure 2E. The graphs are presented as described for Figure 1E, except only categories of information accumulated in Supplementary Table S3 with at least one gene present in the interest group are shown. [file Image_7.TIF]

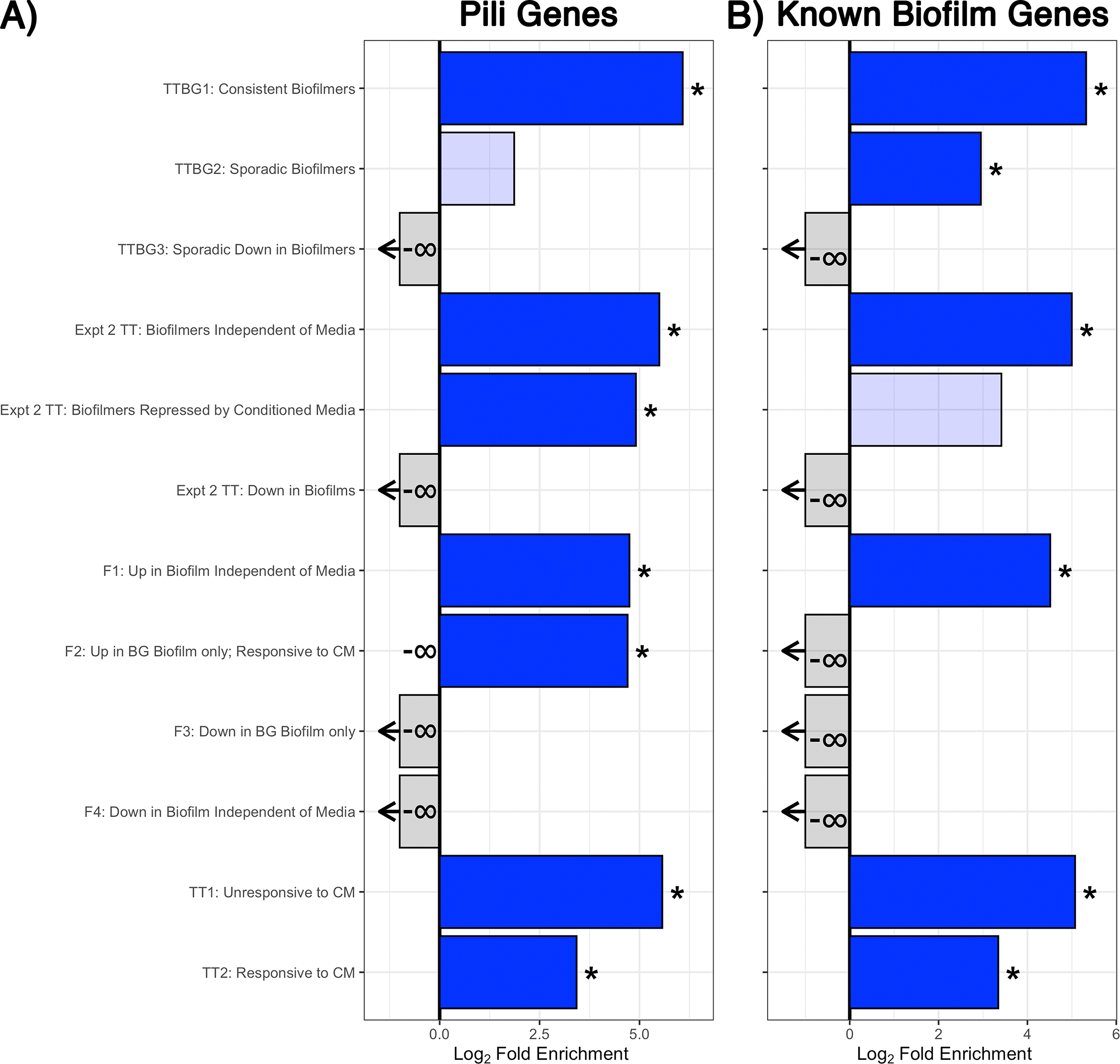

Supplement: Supplementary Figure S8 — Enrichment analyses of (A) pili genes and (B) known biofilm genes in all named RB-Tnseq clusters of interests, as identified in Figures 3, 4 and Supplementary File S4. The graphs are presented as described for Figure 1E. [file Image_8.TIF]

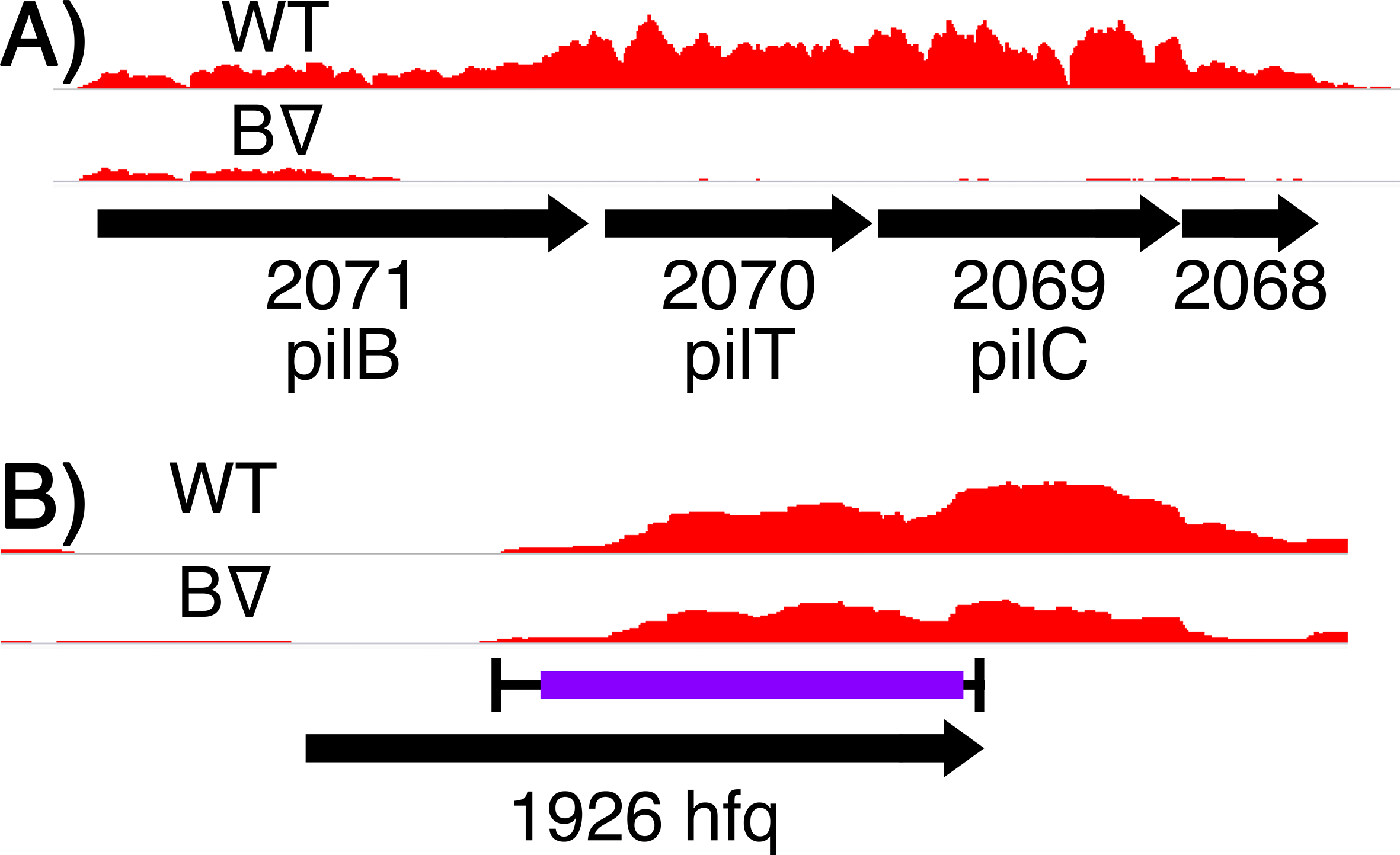

Supplement: Supplementary Figure S9 — RNA-Seq Coverage of pilB operon and hfq. (A) Strand-specific transcriptional coverages in the region of the PilB::Tn5 insertion and the downstream operon in WT and PilB::Tn5 samples in fresh BG-11 on Day 1. Both coverage tracks are at the same scale, with the highest peak being approximately 1,000×. (B) Strand-specific transcriptional coverages in the region of hfq (Synpcc7942_1926) in WT and PilB::Tn5 samples in fresh BG-11 on Day 1. Both coverage tracks are at the same scale, with the highest peak being approximately 70×. Transcriptional coverage starts in the middle of the canonical annotation (black arrow) but before the proposed ORF based on an alternative start codon (black brackets) based on the location of a predicted Sm-like RNA binding domain (purple). [file Image_9.TIF]

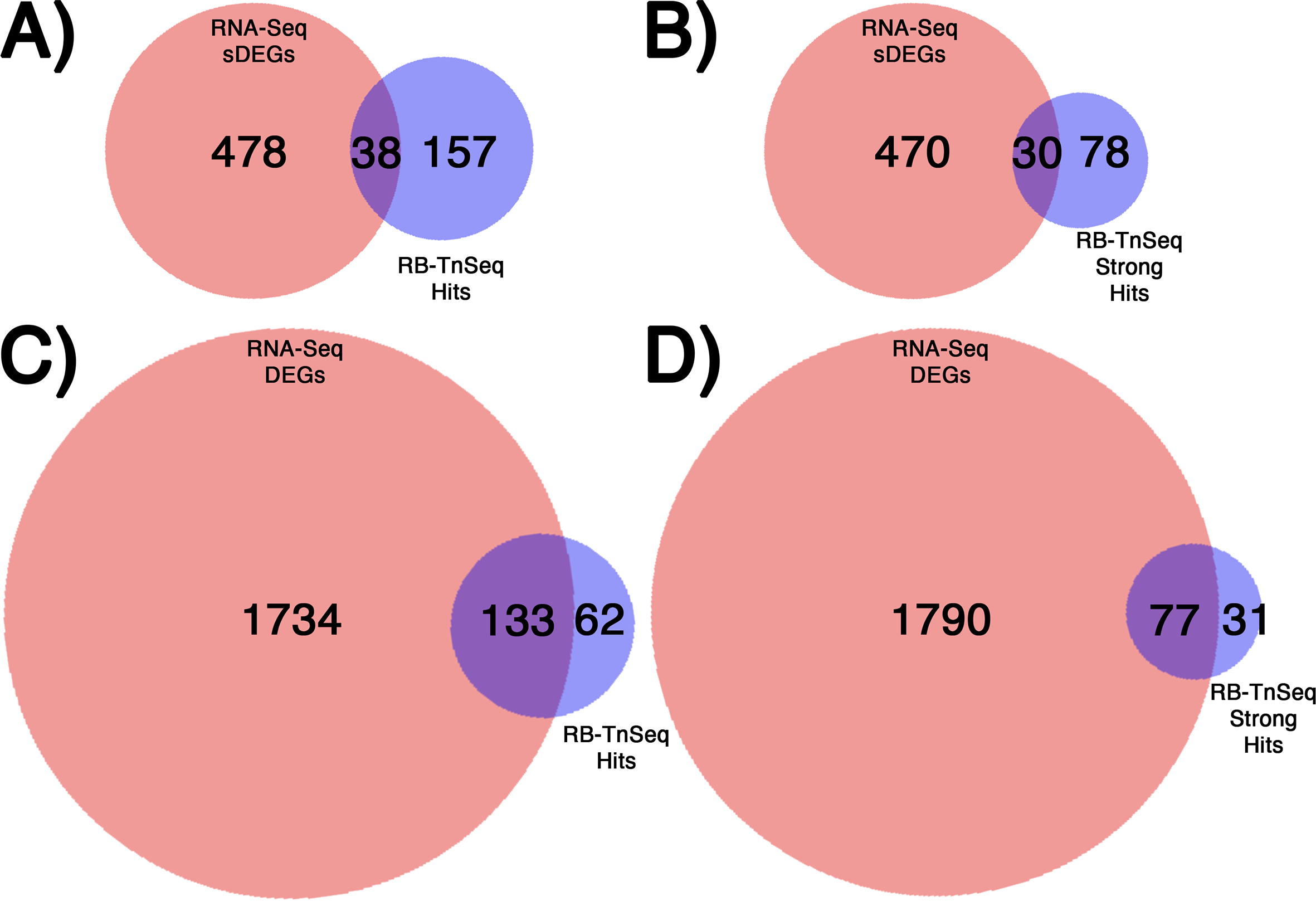

Supplement: Supplementary Figure S10 — Venn Diagrams of Hit Sets for RNA-Seq and RB-TnSeq data sets. (A) Strong DEGs (sDEGs) for RNA-Seq vs. RB-TnSeq Hits, (B) sDEGs for RNA-Seq vs. RB-TnSeq strong hits, (C) DEGs for RNA-Seq vs. RB-TnSeq hits, and (D) DEGs for RNA-Seq vs. RB-TnSeq strong hits. Areas are proportional to numbers provided for overlaps, RNA-Seq only DEGs, or RB-TnSeq hits. [file Image_10.TIF]
